# Tamoxifen Treatment in Hamsters Induces Protection during Taeniosis by *Taenia solium*

**DOI:** 10.1155/2013/280496

**Published:** 2012-12-20

**Authors:** Galileo Escobedo, M. Isabel Palacios-Arreola, Alfonso Olivos, Lorena López-Griego, Jorge Morales-Montor

**Affiliations:** ^1^Unidad de Medicina Experimental, Hospital General de México, México, DF 06726, Mexico; ^2^Departamento de Inmunología, Instituto de Investigaciones Biomédicas, Universidad Nacional Autónoma de México, AP 70228, México, DF 04510, Mexico; ^3^Departamento de Medicina Experimental, Hospital General de México, Facultad de Medicina, Universidad Nacional Autónoma de México, México, DF 06726, Mexico

## Abstract

Human neurocysticercosis by *Taenia solium* is considered an emergent severe brain disorder in developing and developed countries. Discovery of new antiparasitic drugs has been recently aimed to restrain differentiation and establishment of the *T. solium* adult tapeworm, for being considered a central node in the disease propagation to both pigs and humans. Tamoxifen is an antiestrogenic drug with cysticidal action on *Taenia crassiceps*, a close relative of *T. solium*. Thus, we evaluated the effect of tamoxifen on the *in vitro* evagination and the *in vivo* establishment of *T. solium*. *In vitro*, tamoxifen inhibited evagination of *T. solium* cysticerci in a dose-time dependent manner. *In vivo*, administration of tamoxifen to hamsters decreased the intestinal establishment of the parasite by 70%, while recovered tapeworms showed an 80% reduction in length, appearing as scolices without strobilar development. Since tamoxifen did not show any significant effect on the proliferation of antigen-specific immune cells, intestinal inflammation, and expression of Th1/Th2 cytokines in spleen and duodenum, this drug could exert its antiparasite actions by having direct detrimental effects upon the adult tapeworm. These results demonstrate that tamoxifen exhibits a strong cysticidal and antitaeniasic effect on *T. solium* that should be further explored in humans and livestock.

## 1. Introduction

Human neurocysticercosis by *Taenia solium* is considered a serious brain disorder in developing countries [1], with an alarmingly increased number of new cases in developed industrialized nations [2]. Neurocysticercosis has been recently recognized as a major neglected disease in endemic communities of Latin America, with prevalence estimates of infection of 15% for the Mexican population, whereas it increases to 23% and 38% in Ecuador and Honduras, respectively [3]. Furthermore, it has been estimated that around 0.45–1.35 million cases of epilepsy are attributable to neurocysticercosis in those countries, which may directly increase morbidity and mortality rates associated with this parasite infection [3].

The parasite life cycle takes place in both pigs and humans [4]. In this way, pigs develop the intermediate larvae stage of *T. solium*, while the definitive adult tapeworm is found in the human being [5]. After a subject ingests undercooked contaminated pork meat, the *T. solium* larvae starts to differentiate into an adult tapeworm with the ability to establish at the human bowel [5]. Once this tapeworm has developed gravid mature proglottids, thousands of eggs are released with the stools into the environment, where they will be capable to infect free-ranging boars, maintaining the parasite life cycle [1, 5]. In parallel, neurocysticercosis can be acquired by humans once they have been accidentally exposed to stools containing *T. solium* eggs [6]. Thus, the *T. solium* intestinal tapeworm carrier is considered as the central node in the propagation of the disease for both organisms [7]. For this reason, discovery of new anti-taeniosic drugs should be aimed to restrain differentiation, establishment, and egg production of the *T. solium* adult intestinal tapeworm.

 Tamoxifen is a competitive antagonist of the estrogen receptor that has been widely used for treating breast cancer in premenopausal women and gynaecomastia in men receiving hormonal therapy for prostatic carcinoma [8, 9]. Interestingly, the use of this antiestrogenic drug has also proved to be effective against several protozoan parasites, including *Leishmania major*, *L. braziliensis*, *L. chagasi*, *L. amazonensis*, and *Trypanosoma cruzi* [10–13]. Nevertheless, the tamoxifen effect upon helminth cestode parasites has been exclusively studied for the case of *Taenia crassiceps*, the causal agent of experimental murine cysticercosis [14]. Actually, tamoxifen inhibits *T. crassiceps* proliferation and viability *in vitro* [15], whereas it induces protection against the infection *in vivo*, through reducing parasite load by 80% [16]. Since *T. crassiceps* has a very close phylogenetic relationship with *T. solium* [17, 18], we hypothesize that the use of tamoxifen could also exhibit detrimental actions upon the latter one.

 Thus, we evaluated the effect of tamoxifen on *Taenia solium*, focusing on several important aspects of the adult tapeworm stage, including differentiation from cysticercus to worm on *in vitro* cultures, and establishment of the intestinal tapeworm using the hamster model for experimental taeniosis *in vivo*. Our results demonstrate that tamoxifen totally inhibits the *in vitro* evagination of the *Taenia solium* larvae in a dose-response manner, while it also reduces the intestinal establishment of tapeworms by 70%, without affecting the host immune response. This paper could contribute to the search and design of novel therapeutic agents for the control of cysticercosis and taeniosis in livestock and humans.

## 2. Materials and Methods

### 2.1. Ethic Statement

Animal care and experimentation practices at the Instituto de Investigaciones Biomédicas are frequently evaluated by the Institute's Animal Care and Use Committee, in strict accordance with the recommendations in the Guide for the Care and Use of Laboratory Animals of the National Institutes of Health (NIH and The Weatherall Report) of the USA, to ensure compliance with established international regulations and guidelines. The protocol was approved by the Committee on the Ethics of Animal Experiments of the Instituto de Investigaciones Biomédicas, at the Universidad Nacional Autónoma de México (Permit Number: 2009-16). Pigs sacrifice to obtain parasites was performed under sodium pentobarbital anesthesia, and all efforts were made in order to minimize suffering.

### 2.2. Parasites

*T. solium* cysticerci were selected according to the main criteria previously reported by León-Cabrera and coworkers [19]. Briefly, parasites were dissected from the muscle of naturally infected pigs, which were previously euthanized at the Veterinary School of the Universidad Nacional Autónoma de México, under consent of the University Animal Care and Use Committee to ensure compliance with international regulations and guidelines. The fibrous capsule surrounding each cysticercus was carefully separated under a dissection microscope. Once separated, cysticerci were placed in tubes containing sterile PBS (1X) supplemented with 100 U/mL of penicillin-streptomycin-fungizone (Gibco, Grand Island, NY). The tubes were then centrifuged at 1200 rpm/4°C for 10 min and the supernatant was discarded. Cysticerci were then placed in Dulbecco's modified medium (DMEM, Gibco, BRL, Rockville, MD) without fetal calf serum (FCS) supplementation. After this, parasites were washed and centrifuged 3 times at 1200 rpm/4°C for 10 min using DMEM. Afterward, complete and translucent reddish cysticerci were incubated on 6-well culture plates containing DMEM medium supplemented with 25% pig fresh bile for infectivity test. When the evagination rate was higher than 90%, cysticerci were used for subsequent oral infections.

### 2.3. Tamoxifen Concentration-Time Response Curves

All of the *in vitro* cultures were performed using FCS-free DMEM with 100 U/mL of penicillin-streptomycin-fungizone (Gibco, Grand Island, NY). Culture grade tamoxifen was obtained from Sigma (Sigma-Aldrich, USA), and dissolved in ethanol (J. T. Baker) to the desired stock concentration. Stock solutions were sterilized by passage through a 0.2 *μ*M millipore filter and used for culture media supplementation. For concentration-response curves, the experimental design was as follows: 20 parasites equally divided into five culture wells were incubated in presence of 0.01 *μ*M tamoxifen-0.06% ethanol for 20 days; 20 parasites equally divided into five culture wells were incubated in presence of 0.05 *μ*M tamoxifen-0.06% ethanol for 20 days; 20 parasites equally divided into five culture wells were incubated in presence of 0.5 *μ*M tamoxifen-0.06% ethanol for 20 days; finally, 20 parasites equally divided into five culture wells were incubated in presence of 1 *μ*M tamoxifen-0.06% ethanol for 20 days. Control parasites were incubated either in presence of 0.06% ethanol or in absence of this solvent for the same time. For time-response curves, cysticerci were incubated in presence of increasing doses of tamoxifen (0.01 *μ*M, 0.05 *μ*M, 0.5 *μ*M, and 1 *μ*M) for 20 days. Both concentration and time-response experiments were daily inspected for scolex evagination and worm growth using an inverted microscope at 4 and 10X magnification (Olympus, MO21, Tokyo). Worm growth was considered as the millimeter sum of scolex, neck, and strobila, as we previously reported [20]. Cultures were performed under 5% CO_2_ at 37°C, replacing the supplemented culture media every 24 hours during the entire time of the experiments.

### 2.4. Tamoxifen *In Vivo* Administration

Ten female golden hamsters (*Mesocricetus auratus*) of 140–160 g, aging between 8 and 10 weeks, were subcutaneously administered with 1 mg/Kg body weight tamoxifen (Sigma-Aldrich, USA). Each single dose of tamoxifen was diluted in saline solution (0.9% NaCl, J. T. Baker) containing 0.06% ethanol. Two different groups of control animals were used in all of our experiments, as follows: the vehicle group consisted in ten animals subcutaneously administered with saline solution containing 0.06% ethanol; the control group consisted in using ten nonmanipulated animals in order to dismiss a possible effect of manipulation-induced stress on the results. Tamoxifen and vehicle administration was carried out each other day for 4 weeks, in order to maintain a constant serum concentration for the entire time of the experiment. Animals were fed with Purine Diet 5015 (Purine, St. Louis, MO) and water *ad libitum*.

### 2.5. Oral Infection Experiments

Two weeks after the beginning of the drug administration, tamoxifen, vehicle, and control animals were orally infected with four viable *T. solium* cysticerci, according to previous reports [19, 20]. All of the animals were euthanized 15 days postinfection, using a CO_2_-saturated chamber. During animal necropsy, the entire small intestine was dissected and placed on a Petri dish containing sterile PBS (1X) (Sigma-Aldrich, USA). Under a stereoscopic microscope, the lumen of the small intestine was carefully exposed by making a longitudinal cut using a sterile dissection scissor. Duodenum-anchored parasites were then counted and measured with a calibrator. Scolex-associated duodenal tissue was placed in 4% paraformaldehyde (J. T. Baker, México), or Trizol reagent (Invitrogen, Carlsbad, California) for posterior analysis. Immediately after necropsy, mesenteric lymph nodes and spleen were dissected from all of the euthanized animals and placed in RPMI medium-10% FCS (Gibco, BRL, Rockville, MD), or Trizol reagent (Invitrogen, Carlsbad, California), respectively.

### 2.6. Cell Culture and Lymphoid Proliferation

Total leukocytes and red blood cells were individually extracted from the mesenteric lymph nodes of all of the animals. After a single washing with ACK Lysing Buffer (Invitrogen, USA), total leukocytes were recovered and cultured in 96-well sterile plates (1 × 10^4^ cells/well) containing RPMI medium-10% FCS (Gibco-BRL, Rockville, MD), at 37°C in humidified 5% CO_2_ atmosphere for 72 hours. After this time, cultured leukocytes were exposed to 15 *μ*g/well of freshly extracted *T. solium* total antigen during 48 hours. Twenty-four hours before the end of the experiment, 20 *μ*L of AlamarBlue reagent (Biosource International) were added to each culture well. Culture plates were then frozen at −30°C under darkness, and the absorbance was quantified at 570 and 600 nm, using a microplate reader (MultiSkan Ascent, Thermo Scientific). The 570–600 nm lecture coefficient was employed to assess the proliferation index.

### 2.7. Cytokine Expression

Spleen and scolex-associated duodenal tissue were placed in Trizol reagent (Invitrogen, Carlsbad, California). Total RNA extraction was as follows: both tissues were separately disrupted in Trizol reagent (1 mL/0.1 g tissue) and 0.2 mL of chloroform was added per mL of Trizol. The aqueous phase was recovered after 15 min of centrifugation at 13000 rpm, and treated with a same volume of isopropyl alcohol for RNA precipitation. After 15 min of centrifugation at 13000 rpm, the RNA pellet was washed with 75% ethanol and dissolved in RNAase-free water. RNA concentration was determined by absorbance at 260 nm, and its purity was verified after electrophoresis on 1.0% denaturing agarose gel in presence of 2.2 M formaldehyde. Immediately after, total RNA samples were reverse-transcribed by using the M-MLV Retrotranscriptase system and dT primer (Invitrogen, USA). cDNA was then used for specific PCR amplification of IL-4, IL-6, IL-10, IL-12, IFN*γ*, and TNF-*α*, using hamster-specific primers (Table 1) and TaqDNA polymerase in a semiquantitative system (Biotecnologías Universitarias, UNAM, México). Briefly, the 50 *μ*L PCR reaction included 10 *μ*L of previously synthesized cDNA, 5 *μ*L of 10X PCR-buffer (Perkin-Elmer, USA), 1 mM MgCl, 0.2 mM of each dNTP, 0.05 *μ*M of each primer, and 2.5 units of TaqDNA polymerase (Biotecnologias Universitarias, Mexico). After an initial denaturation step at 95°C for 5 min, temperature cycling was as follows: 95°C for 30 s, from 51°C to 62°C (depending on the primer sequence) for 45 s, and 72°C for 45 s during 35 cycles. An extra extension step was completed at 72°C/10 min for each run. The 50 *μ*L of the PCR reaction was electrophoresed on a 2% agarose gel and stained with ethidium bromide in the presence of a 100 bp ladder as molecular weight marker (Gibco, BRL, NY). The relative expression rate of each amplified gene was obtained by optical density analysis (OD), using the 18S-ribosomal RNA as constitutive control of expression.

### 2.8. Histological Examination of Inflammatory Infiltrate

It has been previously reported that hormone-associated factors are able to induce an intestinal inflammatory response associated with *T. solium* tapeworm elimination [21]. We then analyzed a possible tamoxifen-induced intestinal inflammatory response related to control of the parasite load. Scolex-associated duodenal samples from all of the animals were placed in 4% paraformaldehyde for 2 weeks (J. T. Baker, México). After this time, all of the tissues were embedded in paraffin for being posterior cross-sectioned in thin 4 *μ*M slices, by using a microtome (Microtome Olympus Cut 4060, USA). Sections were stained with hematoxylin-eosin for evaluating the inflammatory infiltrate degree on each sample, considered as number of polymorphonuclear leukocytes per ten microvilli, using an optical microscope at 40 and 100x magnification (Nikon Microphot-FXA Microscope).

### 2.9. Statistical Analysis

The *in vitro* and *in vivo* assays were performed in two independent experimental series. Data were pooled and analyzed as mean ± standard deviation using the GraphPad Prism 5 software. After evaluation of the normal distribution of data by means of the Shapiro-Wilk test, one-way analysis of variance (ANOVA), and the Tukey *post-hoc* test were performed to determine significant differences among groups. Differences were considered significant when *P* < 0.05.

## 3. Results

**Figure 1:**
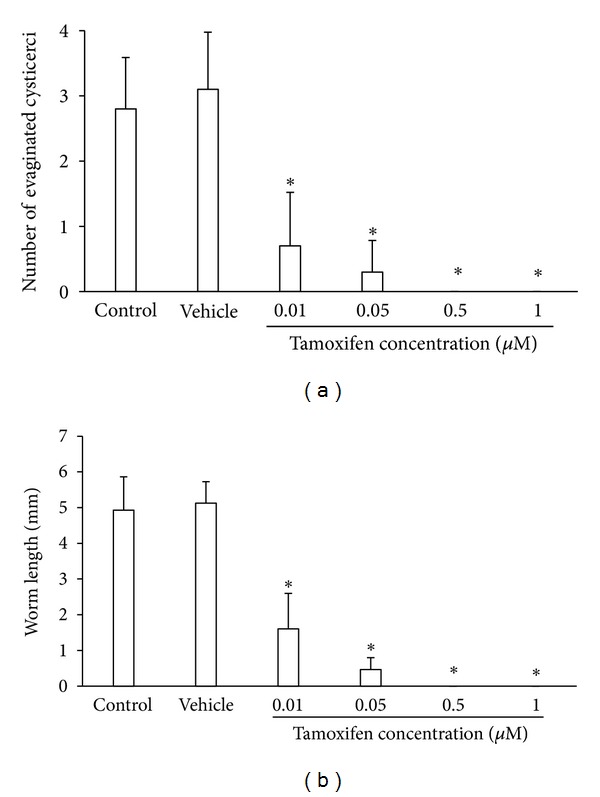
Tamoxifen inhibits the *in vitro* evagination and development of *Taenia solium* cysticerci in a concentration-dependent manner. (a) Concentration-response curves for evaluating the *in vitro* effect of tamoxifen on the evagination of *T. solium* cysticerci in culture. (b) Concentration-response curves for evaluating the effect of tamoxifen on the growth of *in vitro* differentiated *T. solium* worms. Control = parasites cultured in FCS-free DMEM; Vehicle = parasites cultured in FCS-free DMEM containing 0.06% ethanol. Tamoxifen was dissolved in 0.06% ethanol to the desired stock concentration. Total accumulative results at twentieth day of *in vitro* culture are shown. Data were pooled from two independent experiments using cysticerci obtained from two different pigs. Results are presented as mean ± standard deviation. Differences were considered significant when *P* < 0.05. **Significant differences concerning control groups.

Tamoxifen exhibited a strong cysticidal effect on *Taenia solium* larvae *in vitro*. As compared with controls, the use of 0.01 *μ*M tamoxifen decreased parasite evagination by 80%, while increasing concentrations of this antiestrogenic drug totally inhibited differentiation of *in vitro* cultured larvae, reaching a plateau at 0.5 *μ*M after 20 days (Figure 1(a)). Furthermore, the worm length showed a 70% reduction in response to 0.01 *μ*M tamoxifen, whereas no parasite development was observed since 0.5 *μ*M tamoxifen as compared with controls (Figure 1(b)).

**Figure 2:**
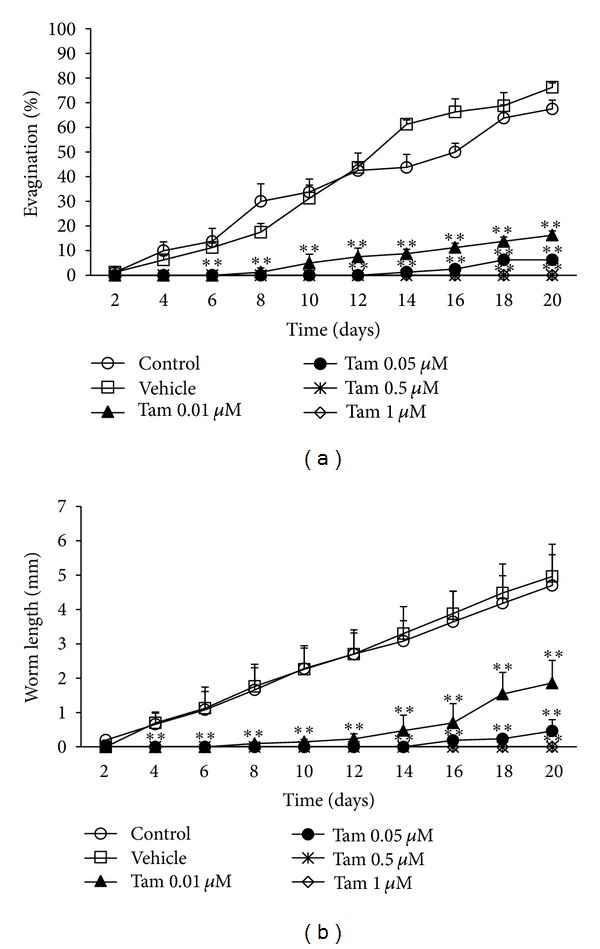
Tamoxifen inhibits the *in vitro* evagination and development of *Taenia solium* cysticerci in a time-dependent manner. (a) Time-response curves for evaluating the *in vitro* effect of tamoxifen on the evagination of *T. solium* cysticerci after 20 days in culture. (b) Time-response curves for evaluating the effect of tamoxifen on the growth of *in vitro* differentiated *T. solium* worms after 20 days in culture. Control = parasites cultured in FCS-free DMEM; Vehicle = parasites cultured in FCS-free DMEM containing 0.06% ethanol. Tamoxifen was dissolved in 0.06% ethanol to the desired stock concentration (Tam). Data were pooled from two independent experiments using cysticerci obtained from two different pigs. Results are presented as mean ± standard deviation. Differences were considered significant when *P* < 0.05. **Significant differences concerning control groups.

Control cysticerci displayed a spontaneous evagination after two days of *in vitro* culture, reaching a plateau at eighteen day (Figure 2(a)). On the contrary, parasites exposed to 0.01 *μ*M tamoxifen started to differentiate after eight days in culture, whereas 0.05 *μ*M tamoxifen delayed this process by double of the time when compared with controls (Figure 2(a)). The *T. solium* scolex evagination was not observed in parasites exposed to 0.5 and 1 *μ*M tamoxifen after 20 days of *in vitro* culture (Figure 2(a)). Similarly, *in vitro* differentiated worms reached a 4.96 ± 0.93 mm length under control conditions, while cysticerci differentiated in presence of the lowest tamoxifen concentration showed a 1.86 ± 0.65 mm maximum length (Figure 2(b)). Once again, increasing concentrations of tamoxifen induced a significant delay in the parasite development onset, accompanied by a progressive diminution in the growth of *in vitro* differentiated worms (Figure 2(b)). Notably, since no difference between control groups were observed, we assume that addition of 0.06% ethanol to the culture media had no significant effects on *T. solium* scolex evagination and worm growth *in vitro* (Figures 1 and 2).

**Figure 3:**
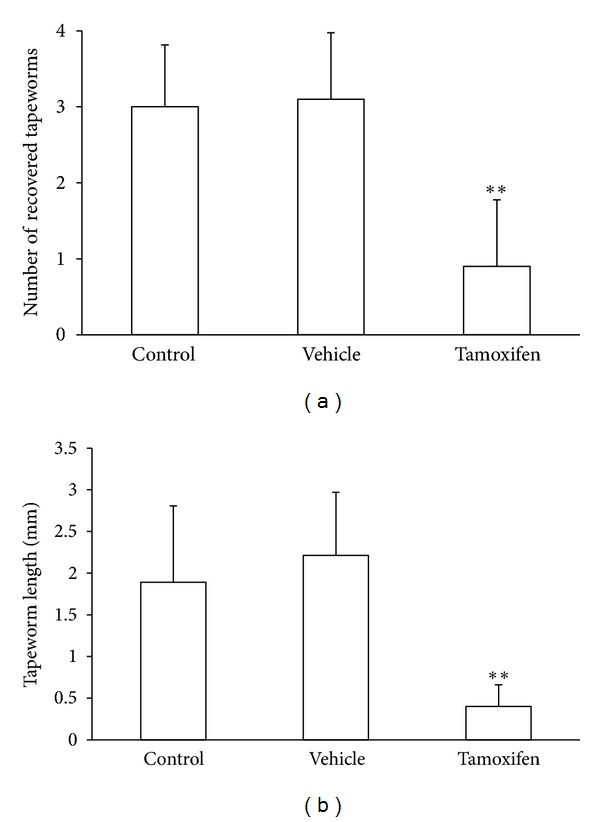
Tamoxifen impairs the *in vivo* establishment of *Taenia solium*. Hamsters were subcutaneously treated with 1 mg/Kg body weight tamoxifen and orally infected using four viable *T. solium* cysticerci each. Control = Nonmanipulated animals, infected with four viable cysticerci each; Vehicle = animals subcutaneously treated with 0.06% ethanol-saline solution, infected with four viable cysticerci each. (a) Evaluation of the number of duodenum-anchored tapeworms at day fifteen post-infection. (b) Assessment of the length of recovered tapeworms at day fifteen post-infection. Data were pooled from two independent experiments using ten animals per group in each experimental series and cysticerci obtained from two different pigs. Results are presented as mean ± standard deviation. Differences were considered significant when *P* < 0.05. **Significant differences concerning control groups.

*In vivo*, tamoxifen exerted a protective effect against the *T. solium* intestinal infection, diminishing parasite load and development. In fact, hamsters treated with this antiestrogenic drug exhibited a significant 70% reduction in the number of duodenum-anchored *T. solium* tapeworms, as compared to controls (Figure 3(a)). Furthermore, while vehicle-treated and control animals had between 3 and 4 viable tapeworms associated to the host duodenal mucosa, tamoxifen-treated hamsters showed no more than 1 or 2 poorly developed parasites (Figure 3(a)). Indeed, tapeworms from both control groups reached a maximum length of 2.21 ± 0.75 mm (Figure 3(b)), exhibiting well differentiated rostellum, suckers, and strobila (data not shown). In contrast, parasites from tamoxifen-treated hamsters did not grow up more than 0.42 ± 0.25 mm in length (Figure 3(b)), frequently appearing as scolices without strobilar development.

**Figure 4:**
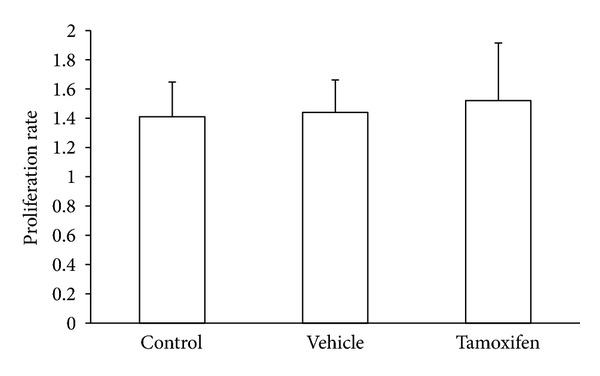
Evaluation of the proliferation rate of antigen-specific immune cells. Total leukocytes from mesenteric lymph nodes of tamoxifen-treated, vehicle-treated, and control hamsters were separately cultured in triplicate in presence of 15 *μ*g/well of *T. solium* total antigen. Proliferation rate was estimated after 48 hours under described conditions. No significant differences in the leukocyte proliferation rate were observed among experimental groups. Control = Nonmanipulated animals, infected with four viable cysticerci each; Vehicle = animals subcutaneously treated with 0.06% ethanol-saline solution, infected with four viable cysticerci each; Tamoxifen = animals subcutaneously treated with 1 mg/Kg BW tamoxifen. Data were pooled from two independent experiments using ten animals per group in each experimental series and cysticerci obtained from two different pigs. Data are expressed as mean ± standard deviation. Differences were considered significant when *P* < 0.05.

In order to determine a possible mechanism through which tamoxifen could exert its protective role during the experimental taeniosis in hamsters, total leukocytes from mesenteric lymph nodes were assayed for antigen-specific proliferation (Figure 4). Interestingly, there were no significant differences in the lymphoid proliferation rate between tamoxifen-treated animals and controls (Figure 4).

**Figure 5:**
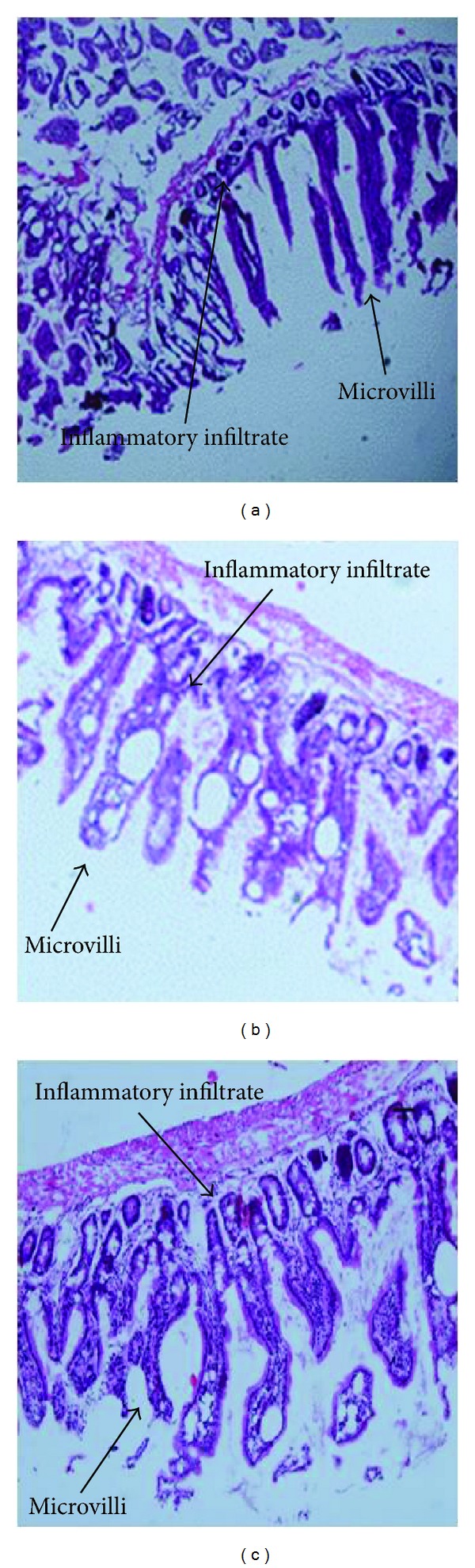
Histological assessment of the duodenal inflammatory infiltrate associated with the *Taenia solium* intestinal infection. Scolex-associated duodenal samples from tamoxifen-treated (c), vehicle-treated (b), and control (a) hamsters were stained with hematoxylin-eosin. The inflammatory infiltrate degree was considered as the number of polymorphonuclear leukocytes per ten microvilli. No significant differences in the inflammatory level of the intestinal mucosa were observed among experimental groups. Data were pooled from two independent experiments using ten animals per group in each experimental series and cysticerci obtained from two different pigs. Differences were considered significant when *P* < 0.05.

 As intestinal inflammation has been related to parasite elimination, we decided to evaluate whether tamoxifen administration is able to induce recruiting of inflammatory cells into the host duodenal mucosa (Figure 5). The duodenal tissue from tamoxifen-treated and control hamsters showed well defined intestinal microvilli on the mucosa, accompanied by a scant inflammatory infiltrate probably associated with parasite attachment (Figure 5). No significant differences in the percent of infiltrated neutrophils, eosinophils, and basophils into the intestinal mucosa of tamoxifen-treated, vehicle-treated, and control animals were observed (Figure 5).

**Figure 6:**
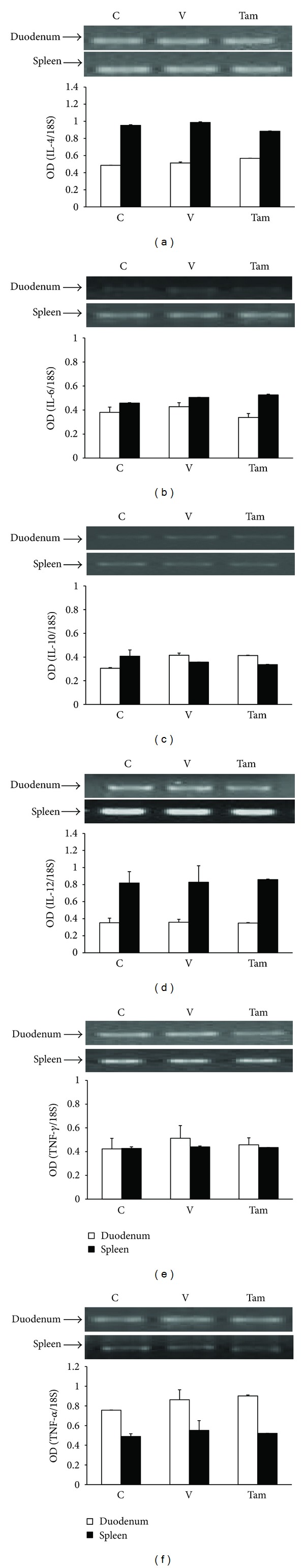
Expression levels of Th1 (a), Th2 (b), proinflammatory (c), and anti-inflammatory cytokines (d) associated with the *Taenia solium* intestinal infection. Cytokine expression in duodenum and spleen tissue samples from tamoxifen-treated, vehicle-treated, and control hamsters was analyzed. An increase in the expression of IL-4, IL-12, IFN-*γ*, and TNF-*α* was strongly associated with the *Taenia solium* intestinal infection. However, no significant changes in this cytokine expression pattern were observed among experimental groups. Control = Nonmanipulated animals, infected with four viable cysticerci each; Vehicle = animals subcutaneously treated with 0.06% ethanol-saline solution, infected with four viable cysticerci each; Tam = animals subcutaneously treated with 1 mg/Kg BW tamoxifen, infected with four viable cysticerci each. Data were pooled from two independent experiments using ten animals per group in each experimental series and cysticerci obtained from two different pigs. Differences were considered significant when *P* < 0.05.

 It has been previously reported that hormone-associated factors can stimulate cytokine expression which in turn is associated with *T. solium* tapeworm elimination. We then studied whether tamoxifen treatment could promote an immunostimulatory effect through inducing cytokine expression at the local and systemic levels. Locally at the duodenum, it was a clear expression of IL-4, IL-12, IFN-*γ*, and TNF-*α* in vehicle-treated and control hamsters (Figure 6). Nevertheless, expression of these cytokines was no significantly changed concerning tamoxifen-treated animals (Figure 6). Systemically at the spleen, the cytokine expression pattern was similar to that observed in the duodenum, characterized by high mRNA levels of IL-4 and IL-12, besides IFN-*γ* and TNF-*α* (Figure 6). However, once again there were not significant differences in the spleen cytokine expression between tamoxifen-treated and control animals (Figure 6).

## 4. Discussion

To our knowledge, this study describes for the first time the effect of tamoxifen upon the *in vitro* evagination and the *in vivo* establishment of *Taenia solium*. Conventional drugs against intestinal taeniosis (such as albendazole, praziquantel, or niclosamide) exhibit numerous side effects in humans, as well as induction of drug-resistant parasite strains. Besides those inconvenient, these antihelminthic drugs have shown to be only effective as therapeutic agents but not in prophylactic schemes. Taking also into consideration that the adult tapeworm carrier has been now recognized as the central node in the maintaining of the disease dissemination to both humans and pigs [1, 2, 7], several research groups have then focused on designing new drugs and vaccines in order to prevent the intestinal establishment of *T. solium*, as a promissory strategy for interrupting the parasite life cycle and possibly the infection [19, 21, 22]. In this sense, the S3PVac synthetic peptide vaccine protects hamsters orally exposed to *T. solium* cysticerci by 74% [22], whereas the use of *T. solium*-derived recombinant proteins seems to confer around 40–100% protection [19]. Our research group recently reported that administration of progesterone to infected hamsters is able to diminish the adult tapeworm establishment by 80% [21]. However, effectiveness of synthetic or recombinant vaccines is known to be dependent on host-associated factors such as host's sex and age, as well as parasite-associated factors including cysticerci size, morphological aspect, and genetic background [19, 23]. Similarly, hormonal therapy with progesterone exhibits controversial results, inducing protection *in vivo* but stimulating parasite evagination and growth *in vitro* [20, 21]. Interestingly, our results suggest that low concentrations of tamoxifen exhibit a strong cysticidal effect upon *T. solium* cysticerci in culture, while administration of this antiestrogenic drug protects hamsters against the intestinal tapeworm establishment. Thus, tamoxifen seems to show consistent results *in vitro* and *in vivo*, which suggests that a possible future antiparasite therapy could not only be restricted to treat the adult tapeworm carrier, but also be extended to pigs in order to diminish the *T. solium* metacestode's viability and differentiation capacity. An additional interesting issue that should be taken into consideration in designing more effective strategies against the adult stage of *T. solium*, is a combinatorial therapy using immunogenic molecules and low doses of tamoxifen. In this sense, the combined use of vaccines with hormone-associated factors has previously shown major results against virus and bacterial infections [24, 25]. We thus considered that such a combinatorial therapy against *T. solium* could improve the protective responses reported to date.

An intriguing question is the possible mechanism through which tamoxifen restricts the *T. solium in vivo* establishment. It has been widely described that hormone-associated factors are able to enhance the host immune response during a parasite infection, as it is well known for murine strongyloidiasis, experimental cysticercosis, trypanosomiasis in rats, murine trichuriasis, and trichinosis in guinea pigs, among many others [21, 26–29]. For the specific case of experimental taeniosis in hamsters, it has been previously reported that an intestinal inflammatory response accompanied by a local expression of Th1 and Th2 cytokines are involved in parasite elimination [21, 30]. However, our data suggest that although tamoxifen induces a strong restrictive response against the *T. solium* adult tapeworm, this effect does not seem to be through recruiting inflammatory cells into the intestinal mucosa, or stimulating the local or systemic expression of IL-4, IL-12, IFN-*γ*, and TNF-*α*. Furthermore, our research group recently showed that proliferation of antigen-specific immune cells could be stimulated by hormone-associated factors and involved in the eradication of *T. solium* [21]. Nevertheless, tamoxifen administration did not have a significant effect on the proliferation of antigen-specific immune cells. In this sense, as we mentioned, a previous study demonstrated that tamoxifen exerts a strong protective effect against experimental cysticercosis in mice by two main mechanisms: induction of the IL-2 expression, and by having direct detrimental effects upon *Taenia crassiceps* viability and reproduction [16]. It has been also reported that tamoxifen is able to directly diminish viability of all life cycle stages of *Trypanosma cruzi* at micromolar concentrations [13]. In a similar way, *Leishmania braziliensis* and *L. chagasi* intracellular amastigotes considerably decrease their viability in response to the *in vitro* treatment with tamoxifen [11]. Since neither humoral immunity nor the cellular response associated with *T. solium* elimination increase in response to tamoxifen treatment, and considering that this drug is able to directly decrease viability in protozoa and helminth parasites, it is then possible that tamoxifen effects described in this paper could not be mediated by the hamster's immune system, but through having direct detrimental actions upon the adult tapeworm of the parasite. This possibility seems to be plausible since the study of the *T. solium* genome sequences revealed the presence of hormone response genes [31], and it has been previously reported that helminth parasites are able to respond to host-derived hormonal factors [15, 32–34]. Additionally, it has been previously described that tamoxifen increases synthesis of nitric oxide (NO) in fibroblasts, and bone marrow-derived macrophages [12, 35]. Thus, in order to elucidate a possible alternative mechanism through which tamoxifen could exert its antitaeniasic properties, it is convenient to assess whether tamoxifen treatment in *T. solium*-infected hamsters is capable of increasing NO release, evaluating the ability of reactive nitrogen species against helminth parasites such as *T. solium*. However, such an intriguing hypothesis and questions require further experimental investigation.

In here, we have described a new cysticidal action of tamoxifen on the helminth cestode *T. solium*. Since collateral effects of high tamoxifen doses have been largely documented in clinical trials, the use of low doses of this drug as a short-term therapy for treating taeniasic individuals may be a novel alternative approach for disrupting the *T. solium* life cycle with minimal secondary effects for the host. Another promissory strategy for some poor communities involves administration of tamoxifen to rural free-ranging pigs for a short period of time, in order to diminish cysticerci viability and potential differentiation into an adult tapeworm in the human being. Collectively, these results could open an interesting window in the discovery of new therapeutic properties of old drugs for the treatment of parasite diseases in humans and livestock.

## Figures and Tables

**Table 1:** Primers used for amplification of hamster-specific genes. Primer sequences were designed based on hamster-specific gene sequences reported in the Gene databank, NCBI, NIH. Primer sequence as well as molecular weight expected of the PCR product is shown.

Primer definition	Primer sequence	Molecular weight of the PCR product (bp)
IL-4 Forward	5′-CCAGGTCACAGAAAAAGGGA-3′	247
IL-4 Reverse	5′-CGTGGACTCATTCACATTGC-3′	
IL-6 Forward	5′-CAACAAGTCGGAGGTTTGGT-3′	302
IL-6 Reverse	5′-AGGGTTTTGATGGTGCTCTG-3′	
IL-10 Forward	5′-CTGACTCCTTACTGCAGGACT-3′	267
IL-10 Reverse	5′-TGAAGACGCCTTTCTCTTGG-3′	
IL-12 Forward	5′-CTCTGAGCCACTCACGA-3′	167
IL-12 Reverse	5′-GTCAGTGCTGATTGCA-3′	
IFN-*γ* Forward	5′-CAAAAGGCTGGTGACACAAA-3′	326
IFN-*γ* Reverse	5′-TTCTTGTTGGGACGATTTCC-3′	
TNF-*α* Forward	5′-GGGAAGAGAAGTTCCCCAAC-3′	229
TNF-*α* Reverse	5′-TAAACCAGGTACAGCCCGTC-3′	
18S Forward	5′-CGCGGTTCTATTTTGTTGGT-3′	219
18S Reverse	5′-AGTCGGCATCGTTTATGGTC-3′	

bp: base pairs.
